# Fermentative production of enantiopure (*S*)-linalool using a metabolically engineered *Pantoea ananatis*

**DOI:** 10.1186/s12934-021-01543-0

**Published:** 2021-03-02

**Authors:** Nobuhisa Nitta, Yoshinori Tajima, Yoko Yamamoto, Mika Moriya, Akiko Matsudaira, Yasushi Hoshino, Yousuke Nishio, Yoshihiro Usuda

**Affiliations:** Research Institute for Bioscience Products & Fine Chemicals, Ajinomoto Co., Inc., Kawasaki, Japan

**Keywords:** Linalool, Biphasic fermentation, Mevalonate pathway, Solubility tag, *Pantoea ananatis*

## Abstract

**Background:**

Linalool, an acyclic monoterpene alcohol, is extensively used in the flavor and fragrance industries and exists as two enantiomers, (*S*)- and (*R*)-linalool, which have different odors and biological properties. Linalool extraction from natural plant tissues suffers from low product yield. Although linalool can also be chemically synthesized, its enantioselective production is difficult. Microbial production of terpenes has recently emerged as a novel, environmental-friendly alternative. Stereoselective production can also be achieved using this approach via enzymatic reactions. We previously succeeded in producing enantiopure (*S*)-linalool using a metabolically engineered *Pantoea ananatis*, a member of the Enterobacteriaceae family of bacteria, via the heterologous mevalonate pathway with the highest linalool titer ever reported from engineered microbes.

**Results:**

Here, we genetically modified a previously developed *P. ananatis* strain expressing the (*S*)-linalool synthase (AaLINS) from *Actinidia arguta* to further improve (*S*)-linalool production. AaLINS was mostly expressed as an insoluble form in *P. ananatis*; its soluble expression level was increased by N-terminal fusion of a halophilic β-lactamase from *Chromohalobacter* sp. 560 with hexahistidine. Furthermore, in combination with elevation of the precursor supply via the mevalonate pathway, the (*S*)-linalool titer was increased approximately 1.4-fold (4.7 ± 0.3 g/L) in comparison with the original strain (3.4 ± 0.2 g/L) in test-tube cultivation with an aqueous-organic biphasic fermentation system using isopropyl myristate as the organic solvent for in situ extraction of cytotoxic and semi-volatile (*S*)-linalool. The most productive strain, IP04S/pBLAAaLINS-ispA*, produced 10.9 g/L of (*S*)-linalool in “dual-phase” fed-batch fermentation, which was divided into a growth-phase and a subsequent production-phase. Thus far, this is the highest reported titer in the production of not only linalool but also all monoterpenes using microbes.

**Conclusions:**

This study demonstrates the potential of our metabolically engineered *P. ananatis* strain as a platform for economically feasible (*S*)-linalool production and provides insights into the stereoselective production of terpenes with high efficiency. This system is an environmentally friendly and economically valuable (*S*)-linalool production alternative. Mass production of enantiopure (*S*)-linalool can also lead to accurate assessment of its biological properties by providing an enantiopure substrate for study.

**Supplementary Information:**

The online version contains supplementary material available at 10.1186/s12934-021-01543-0.

## Background

Terpenes are one of the most abundant classes of natural products with diverse structures and functions (over 50,000 known compounds) and have been widely used as pharmaceuticals, fragrances, and flavors. In plants, terpenes are synthesized by terpene synthases (TPSs) from basic five-carbon precursor units, isopentenyl pyrophosphate (IPP) and dimethylallyl pyrophosphate (DMAPP), which are supplied from either the methylerythritol phosphate pathway or mevalonate (MVA) pathway [1]. Microbial terpene production has recently emerged as an ecologically friendly alternative to extraction from natural vegetations (wood and leaf-derived essential oils), which tends to suffer from low product yield [2, 3]. Fermentation products from natural resources are also more economically valuable in the flavor market than their chemically synthesized counterparts because they can be labeled as “natural”, aligning with emerging consumer preferences for natural substances [4, 5]. Additionally, microbial production allows for enantioselective production of terpenes, which is difficult via chemical synthesis, by exploiting the stereoselectivity (enantioselectivity) of enzymatic reactions such as enantiopure production of (*R*)-α-ionone [5] and ( −)-α-bisabolol [6] in *Escherichia coli*. However, examples of fermentative terpene production reaching 10 g/L titer have been confined to four molecules, viridiflorol, amorpha-4,11-diene [7], β-farnesene [8] (sesquiterpenes), and isoprene (a hemiterpene) [9], according to our literature search [10].

Linalool, an acyclic monoterpene alcohol, has been widely used as a flavor additive and fragrance ingredient [11]. Linalool is synthesized by linalool synthases from geranyl pyrophosphate (GPP), which is generated by the condensation of IPP and DMAPP by GPP synthase in plants. Linalool exists as two enantiomers, (*S*)- and (*R*)-linalool, which are differentiated by the chiral properties of the hydroxylated third carbon; the different enantiomers show distinct odors and biological properties [11]. Since commercially available linalool is mainly racemate or (*R*)-linalool, enantiopure (*S*)-linalool is attractive to the flavor and fragrance industries. Enantiopure (*S*)-linalool production has already been reported in *Saccharomyces cerevisiae* [12] and *Yarrowia lipolytica* [13] expressing (*S*)-linalool synthase (AaLINS) from *Actinida arguta* [14]. We also successfully produced (*S*)-linalool in the cyanobacterium *Synechocystis* sp. PCC 6803 (*Synechocystis*) [15] by co-expressing AaLINS and the S80F mutant of farnesyl pyrophosphate synthase (IspA*; from *E. coli*, functioning as GPP synthase) [16]. However, production levels in these systems range from 240 µg/L to 11.6 mg/L, which cannot meet industrial needs.

*Pantoea ananatis*, a Gram‐negative and yellow‐pigmented bacterium, was identified in 1928 and has been mainly studied as a plant pathogen [17]. In the mid-1990s, a nonpathogenic *P. ananatis* strain AJ13355 was isolated by specialists from Ajinomoto Co., Inc. and has been demonstrated to be an excellent host for L-glutamate production because of its capability to grow at an acidic pH and resist the effects of high concentrations of L-glutamate [18]. The well-developed genetic tools [19, 20] and sequenced complete genome [18] has broadened the attractiveness of *P. ananatis* as a production host for various bio-based materials such as cysteine [21], dicarboxylic acids [22], and isoprene [23]. We constructed a metabolically engineered *P. ananatis* strain named SWITCH-PphoC, which contains heterologous genes of the MVA pathway (*mvaE* and *mvaS* from *Enterococcus faecalis*; MVA kinase gene from *Methanocella paludicola* [*mvk*]; phosphomevalonate kinase, diphosphomevalonate decarboxylase, and IPP isomerase genes from *S. cerevisiae*), to supply IPP/DMAPP for isoprene production [23]. This strain was designed to direct carbon flux to the MVA pathway only under external inorganic phosphate (P_i_)-starved conditions by using the P_i_-starvation-inducible *phoC* promoter for driving the expression of the *mvaES* operon, which encodes the enzymes that catalyze the conversion of acetyl-coenzyme A (CoA) to MVA. Furthermore, enantiopure production of both (*S*)- and (*R*)-linalool at a titer of greater than 1 g/L has been successful with a SWITCH-PphoC strain expressing IspA* and either AaLINS or a (*R*)-linalool synthase from *Streptomyces clavuligerus* [24], whose genes were optimized based on the codon preference of *Synechocystis*, under an aqueous-organic biphasic fermentation system in which monoterpene’s cytotoxicity and product loss by its air-stripping were alleviated [25] using isopropyl myristate (IPM) as an organic solvent [26]. Additionally, the (*S*)-linalool titer was increased by deleting *gcd* (locus_tag PAJ_3473) encoding a glucose dehydrogenase in the SWITCH-PphoC strain (SWITCH-PphoC Δ*gcd*) [26].

In this study, we chose (*S*)-linalool as the target product and aimed to further improve (*S*)-linalool production through several approaches: (1) by increasing carbon flux to IPP/DMAPP from acetyl-CoA through enhancement of the upper component of the MVA pathway; (2) by increasing intracellular AaLINS activity with N-terminal fusion of a halophilic β-lactamase (BLA) from *Chromohalobacter* sp. 560 [27] joined to a hexahistidine (6×His); and (3) by adopting an external P_i_-dependent so-called “dual-phase” fed-batch fermentation [23], which separates the growth-phase from a subsequent production-phase to increase efficiency [28]. Guided by these approaches, the (S)-linalool titer finally reached 10.9 g/L with a 5.1% [w/w] yield from glucose in the biphasic fermentation system, which demonstrates the potential for industrial-scale enantiopure (*S*)-linalool production using *P. ananatis*.

## Results

### Search for putative rate-limiting reaction to increase (*S*)-linalool productivity in SWITCH-PphoC Δ*gcd*/pAaLINS-ispA* strain

**Fig. 1:**
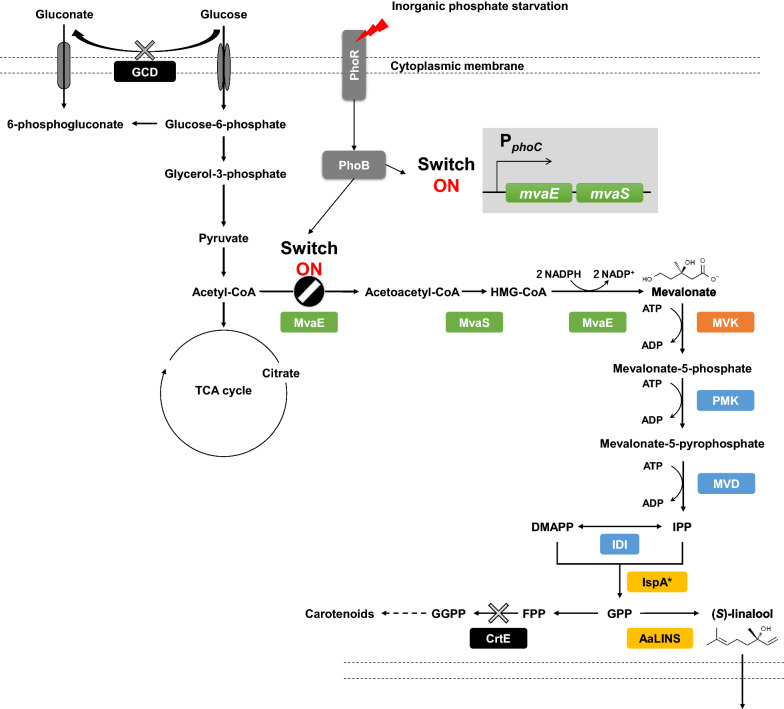
Engineered (*S*)-linalool biosynthetic pathway in *P. ananatis*. Intermediates: DMAPP, dimethylallyl pyrophosphate; FPP, farnesyl pyrophosphate; GGPP, geranylgeranyl pyrophosphate; GPP, geranyl pyrophosphate; HMG-CoA, 3-hydroxy-3-methylglutaryl coenzyme A; IPP, isopentenyl pyrophosphate. Genes and enzymes: AaLINS, (*S*)-linalool synthase; CrtE, GGPP synthase; IDI, IPP isomerase; IspA*, GPP synthase; GCD, membrane-bound glucose dehydrogenase; MvaE, acetoacetyl-CoA thiolase and HMG-CoA reductase; MvaS, HMG-CoA synthase; MVD, mevalonate pyrophosphate decarboxylase; MVK, mevalonate kinase; PhoB, phosphate regulon transcriptional regulatory protein; PhoR, phosphate regulon sensor protein; PMK, phosphomevalonate kinase

The previously constructed (*S*)-linalool-producing strain (SWITCH-PphoC Δ*gcd*/pAaLINS-ispA*) was generated by integrating pACYC177-P_*tac*_-*AaLINS*-ispA* (pAaLINS-ispA*) [15], a co-expression plasmid for AaLINS and ispA*, into the strain SWITCH-PphoC Δ*gcd* [26], which is equipped with genes comprising the whole MVA pathway (Fig. 1). This strain also has a metabolic switch to allow redirection of carbon flux to (*S*)-linalool via the MVA pathway by sensing P_i_-starvation with the PhoB/PhoR two-component system [23]. As a result of test-tube cultivation under P_i_-starved conditions using a biphasic fermentation system, SWITCH-PphoC Δ*gcd*/pAaLINS-ispA* produced 3.4 ± 0.2 g/L of (*S*)-linalool from 60 ± 0.0 g/L of glucose in 48 h (Table 1), which was greater than the reported linalool titers from yeasts [12, 13, 29, 30] and bacteria [15, 31]. As our previous study showed that additional integration of an *mvaES* operon expression-cassette led to higher productivity in an isoprene-producing *P. ananatis* strain [32], the strain IP03 was constructed by integrating one *mvaES* operon expression-cassette into the genome of SWITCH-PphoC Δ*gcd*. The strain IP04 was also constructed from IP03 by integrating one *mvk* expression-cassette to increase carbon flux to IPP/DMAPP. However, strains IP03 and IP04 harboring pAaLINS-ispA* (IP03/pAaLINS-ispA* and IP04/pAaLINS-ispA*) showed lower sugar consumption and (*S*)-linalool productivity than SWITCH-PphoC Δ*gcd*/pAaLINS-ispA* in test-tube cultivation. IP03/pAaLINS-ispA* and IP04/pAaLINS-ispA* were unable to completely consume the initial glucose in 48 h (43 ± 0.6 and 36 ± 0.6 g/L), unlike SWITCH-PphoC Δ*gcd*/pAaLINS-ispA*, and consequently produced only 0.8 ± 0.0 and 1.0 ± 0.0 g/L of (*S*)-linalool, respectively (Table 1). This result indicates that their lower sugar consumptions may result from intracellular accumulation of cytotoxic intermediates such as IPP and DMAPP [7, 33]. This assumption was supported by the observation that SWITCH-PphoC Δ*gcd* and IP04 harboring the empty vector (pACYC177), which are likely to accumulate IPP/DMAPP intracellularly because of the lack of linalool synthase, were unable to completely consume all initial glucose in 48 h (43 ± 3.3 and 38 ± 1.7 g/L, Table 1). These results indicate that a rate-limiting step in (*S*)-linalool production exists among the reactions from IPP/DMAPP to (*S*)-linalool.

**Table 1:** (*S*)-Linalool production in test-tube cultivation

Host strain	Plasmid	(*S*)-linalool (g/L)^a^	Consumed glucose (g/L)^a^	Yield (%)^b^
SWITCH-PphoC Δ*gcd*	pACYC177	ND^c^	43 ± 3.3	–
SWITCH-PphoC Δ*gcd*	pAaLINS-ispA*	3.4 ± 0.2	60 ± 0.0	5.6 ± 0.3
SWITCH-PphoC Δ*gcd*	pBLAAaLINS-ispA*	3.0 ± 0.0	60 ± 0.0	5.0 ± 0.0
IP03	pAaLINS-ispA*	0.8 ± 0.0	43 ± 0.6	1.9 ± 0.0
IP03	pBLAAaLINS-ispA*	4.0 ± 0.2	60 ± 0.0	6.6 ± 0.4
IP04	pACYC177	ND	38 ± 1.7	–
IP04	pAaLINS-ispA*	1.0 ± 0.0	36 ± 0.6	2.8 ± 0.1
IP04	pBLAAaLINS-ispA*	4.7 ± 0.3	60 ± 0.0	7.9 ± 0.2

All strains were cultivated for 48 h under biphasic fermentation using isopropyl myristate. Data are expressed as the mean ± SD of at least three biological replicates

^a^Glucose and (*S*)-linalool concentrations are represented by dividing the total amounts by the volume of aqueous culture

^b^Yield was calculated as grams of product per grams of consumed glucose and is expressed as a percentage. Carbon sources contained in 2 g/L of Bacto yeast extract was not considered in this calculation

^c^ND, Not detected

### Approaches to improve intracellular AaLINS activity

Since the lower sugar consumption in IP04/pAaLINS-ispA* was thought to be attributed to isoprenoid precursor toxicity, which has been reported to be relieved by enhanced TPS activity [34], AaLINS activity was suspected of being a potential bottleneck in (*S*)-linalool production. Several TPSs have been identified as primary bottlenecks in terpene biosynthesis because of their poor in vivo properties [35, 36]. In the experiments described above, *AaLINS* and ispA*, which were codon-optimized for *Synechocystis* [15], were used even in *P. ananatis*; however, the codon usages of heterologous eukaryotic genes are commonly optimized for the prokaryotic host [6, 7, 31, 36] in order to improve their translation rate or efficiency. Therefore, the plasmid pAaLINS_pa-ispA*_pa expressing *AaLINS_pa* and ispA*_pa which were optimized to match the codon-preference of *P. ananatis* was constructed to increase AaLINS production or activity (Additional file 1: Figure S1 and S2). To confirm whether the expression level of AaLINS was increased by synonymous substitution of codons of *AaLINS* and ispA*, sodium dodecyl sulfate–polyacrylamide gel electrophoresis (SDS–PAGE) was conducted with samples of both SWITCH-PphoC Δ*gcd*/pAaLINS-ispA* and SWITCH-PphoC Δ*gcd*/pAaLINS_pa-ispA*_pa. As a result, a putative band of AaLINS (molecular mass: 63 kDa) was observed in the crude homogenate containing soluble and insoluble proteins, whereas the band of AaLINS_pa was not visible even in the crude homogenate (Additional file 1: Figure S3). Contrary to our expectation, these data revealed that the total expression level of AaLINS_pa was lower than that of AaLINS, even after the codon-optimization for *P. ananatis*. Consistently, strain SWITCH-PphoC Δ*gcd*/pAaLINS_pa-ispA*_pa produced only 13 ± 1.2 mg/L of (*S*)-linalool in test-tube cultivation (Additional file 1: Table S1), indicating that codon-optimization of *AaLINS* and ispA* for *Synechocystis* unexpectedly led to higher AaLINS expression and (*S*)-linalool titers.

**Fig. 2:**
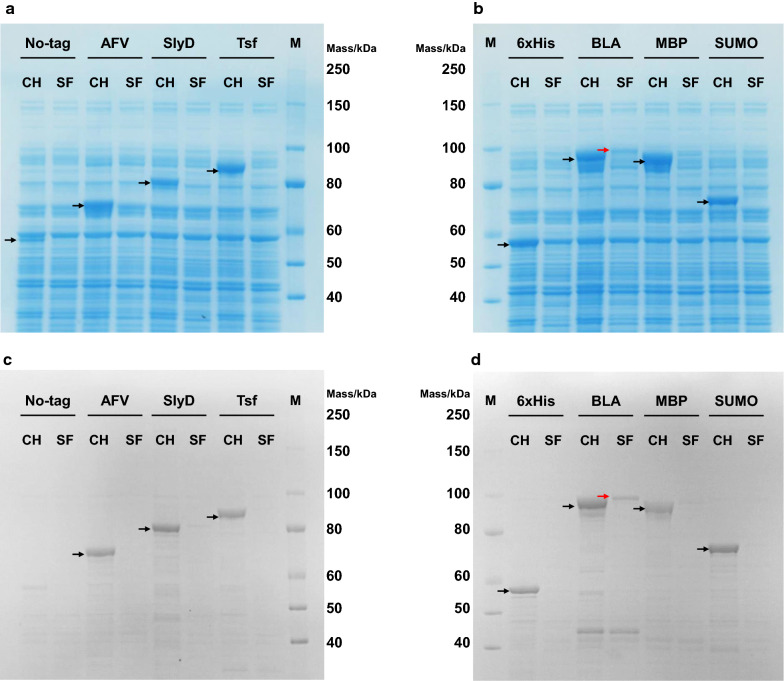
SDS–PAGE gels illustrating total and soluble expression levels of solubility-tag fused AaLINS variants. **a**, **b** Gels stained with Coomassie Brilliant Blue; **c**, **d** Gels stained with an anti-polyhistidine label (a fluorescent dye conjugated to nickel-nitrilotriacetic acid complex). CH, SF, and M denote crude homogenate, soluble fraction, and protein standard, respectively. Samples were prepared from SC17(0) harboring each pSol plasmid grown in LB medium containing rhamnose. Each applied sample contained 10 µg of soluble proteins. Arrow shows each AaLINS variant. AFV, AFV1–99 protein from Acidianus filamentous virus 1; BLA, halophilic β-lactamase from *Chromohalobacter* sp. 560; 6×His, hexahistidine; MBP, maltose binding protein; SlyD, FKBP-type peptidyl-prolyl *cis–trans* isomerase; SUMO, small ubiquitin-like modifier; Tsf, *E. coli* elongation factor

Meanwhile, SDS–PAGE revealed that AaLINS was mostly expressed as insoluble forms in *P. ananatis*; the band of AaLINS was mainly detected in the insoluble fraction (Additional file 1: Figure S3). Specific solubility-tag fusion to the N-terminus of the TPS improves its solubility in *E. coli*, as was found with fusion of the small ubiquitin-like modifier (SUMO) to the ( +)-zizaene synthase from *Chrysopogon zizanioides* [37], and of the maltose binding protein (MBP) to the valencene synthase from *Callitropsis nootkatensis* [38]. Therefore, the N-terminal solubility-tag fusion approach was adopted for AaLINS to increase its soluble expression level. To identify an effective fusion partner protein for AaLINS, six solubility-tags joined to a 6×His (AFV1–99 protein from Acidianus filamentous virus 1, BLA, MBP, FKBP-type peptidyl-prolyl *cis–trans* isomerase, SUMO, and an *E. coli* elongation factor) were evaluated with the *P. ananatis* SC17(0) strain [19] using ready-to-use pSol vectors [39]. Each of the six constructed strains expressed AaLINS fused with one of six solubility-tags under control of a rhamnose-inducible promoter. Control strains capable of expressing untagged AaLINS or a 6×His-tagged AaLINS were also constructed (SC17(0)/pSol-AaLINS and SC17(0)/pSol-HisAaLINS). The results of SDS–PAGE reconfirmed that AaLINS was mostly expressed as insoluble forms in *P. ananatis*. Bands of 6×His-tagged AaLINS and untagged AaLINS were not visible in their soluble protein fractions, whereas they were observed in the crude homogenates (Fig. 2). A faint band appeared at approximately 60 kDa in the untagged control lane in the gel stained with anti-polyhistidine label, which was non-specific (Fig. 2c). In contrast, a band of 6×His-BLA-fused AaLINS (molecular mass: 105 kDa) was observed in the soluble protein fraction by both Coomassie Brilliant Blue (CBB) staining and fluorescence staining of His-tagged proteins (Fig. 2b, d). The difference in the migration of 6×His-BLA-fused AaLINS between the crude homogenate and soluble fraction may have occurred due to overloading of AaLINS derived from the insoluble fraction. Fusion of other evaluated solubility-tags did not improve the solubility from baseline or showed a lower degree of improvement than the 6×His-BLA-fusion (Fig. 2). Aside from solubility, all seven AaLINS variants fused with each solubility-tag appeared to show higher total (insoluble and soluble forms) expression level than untagged AaLINS (Fig. 2a, b). These results indicate that the N-terminal 6×His-tag fusion itself influences the total AaLINS expression level and demonstrate that fusing 6×His-BLA to AaLINS is the most promising means of increasing its intracellular expression level, solubility, and activity.

### Biotransformation assay with 6×His-BLA-fused AaLINS

**Fig. 3:**
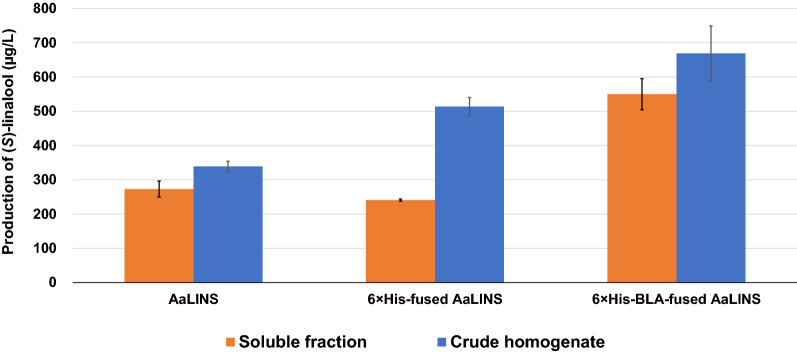
In vitro biotransformation assay with samples prepared from SC17(0)/pSol-AaLINS, SC17(0)/pSol-HisAaLINS, and SC17(0)/pSol-BLAAaLINS. The strains were grown in LB medium containing rhamnose. Each reaction mixture contained 300 mg/L of soluble proteins. Data represent the average of three biological replicates, and error bars represent the standard deviation

An in vitro biotransformation assay was conducted to confirm whether 6×His-BLA-fused AaLINS still retained the capability of converting GPP to (*S*)-linalool, and whether intracellular AaLINS activity could be increased by 6×His-BLA-fusion. The assay consisted of adding crude homogenate or soluble protein fraction of SC17(0)/pSol-AaLINS, SC17(0)/pSol-HisAaLINS, and SC17(0)/pSol-BLAAaLINS to the reaction mixture containing the substrate (GPP) and cofactor (Mg^2+^), respectively. After a 26-h reaction at 30 °C, 273 ± 23, 241 ± 3, and 550 ± 45 µg/L of (*S*)-linalool was detected in the reaction buffer containing the soluble protein fraction of SC17(0)/pSol-AaLINS, SC17(0)/pSol-HisAaLINS, and SC17(0)/pSol-BLAAaLINS, respectively (Fig. 3). When the crude homogenates were used for the assay, 339 ± 15, 514 ± 26, and 669 ± 80 µg/L of (*S*)-linalool was detected in the reaction buffer containing samples of SC17(0)/pSol-AaLINS, SC17(0)/pSol-HisAaLINS, and SC17(0)/pSol-BLAAaLINS, respectively (Fig. 3). This result shows that fusing only 6×His-tag to AaLINS increased its total activity despite nearly no solubility enhancement compared to untagged AaLINS (Fig. 2), which was supported by the result that the (*S*)-linalool titer of strain SWITCH-PphoC Δ*gcd* expressing IspA*_pa and 6×His-tagged AaLINS_pa (306 ± 49 mg/L) was approximately 30 times higher than that of SWITCH-PphoC Δ*gcd*/pAaLINS_pa-ispA*_pa (Additional file 1: Table S1). These data reveal that even with the 6×His-BLA-moiety attached, the (*S*)-linalool producing capability was maintained, and that N-terminal fusion of 6×His-BLA increased the total intracellular AaLINS activity.

### Collective impact of 6×His-BLA-fusion to AaLINS and enhancement of the MVA pathway on (*S*)-linalool production

To evaluate the impact of 6×His-BLA-fusion to AaLINS on (*S*)-linalool production, strains SWITCH-PphoC Δ*gcd*, IP03, and IP04 harboring pBLAAaLINS-ispA*, a plasmid for over-expressing IspA* and 6×His-BLA-fused AaLINS, were cultured in test tubes. IP03/pBLAAaLINS-ispA* and IP04/pBLAAaLINS-ispA* produced 4.0 ± 0.2 g/L of (*S*)-linalool with a 6.6 ± 0.4% yield and 4.7 ± 0.3 g/L of (*S*)-linalool with a 7.9 ± 0.2% yield, respectively (Table 1). Both IP03/pBLAAaLINS-ispA* and IP04/pBLAAaLINS-ispA* completely consumed the initial glucose, unlike IP03/pAaLINS-ispA* and IP04/pAaLINS-ispA*. These data indicate that the bottleneck of (*S*)-linalool production in IP04/pAaLINS-ispA*, which expressed untagged AaLINS, was intracellular AaLINS activity. In contrast, SWITCH-PphoC Δ*gcd*/pBLAAaLINS-ispA* produced only 3.0 ± 0.0 g/L of (S)-linalool, which was almost the same as that produced by SWITCH-PphoC Δ*gcd*/pAaLINS-ispA* (3.4 ± 0.2 g/L) (Table 1). Therefore, the putative bottleneck for (*S*)-linalool production in SWITCH-PphoC Δ*gcd*/pBLAAaLINS-ispA* was the supply of IPP/DMAPP or GPP, and this bottleneck was eliminated by the additional integration of the *mvaES* operon and *mvk* expression cassette in a step-by-step manner. As a result of the collective effects of both the higher intracellular AaLINS activity imparted by fusion to 6×His-BLA and higher GPP supply by enhancement of the MVA pathway, the (*S*)-linalool titer of IP04/pBLAAaLINS-ispA* was approximately 1.4-fold greater than that of SWITCH-PphoC Δ*gcd*/pAaLINS-ispA*.

**Fig. 4:**
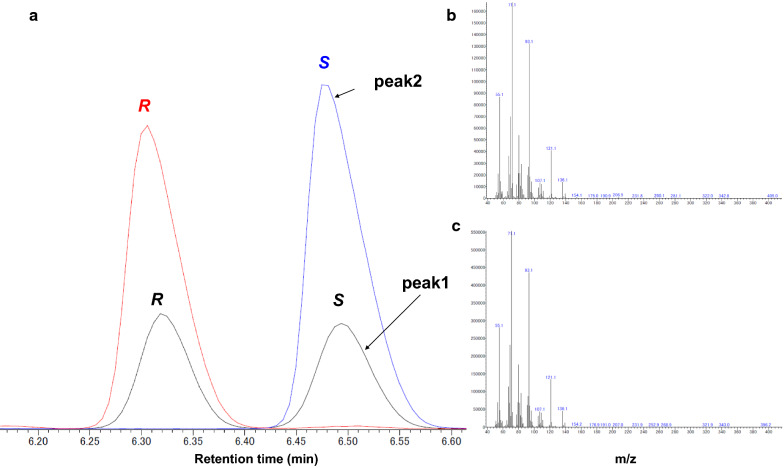
Identification of the absolute configuration of linalool. **a** GC–MS profiles with a chiral column. Black line, standard of racemic linalool; Red line, standard of (*R*)-linalool; Blue line, linalool produced by SWITCH-PphoCΔ*gcd*/pBLAAaLINS-ispA*. **b** Mass spectrum of the peak of (*S*)-linalool in the racemic linalool reagent, which is indicated with black arrow (peak 1). **c** Mass spectrum of the peak of linalool produced by strain SWITCH-PphoC Δ*gcd*/pBLAAaLINS-ispA*, indicated with blue arrow (peak 2)

In addition, to confirm whether the linalool synthesized from GPP by 6×His-BLA-fused AaLINS was still exclusively (*S*)-enantiomer, the culture sample of SWITCH-PphoC Δ*gcd*/pBLAAaLINS-ispA* was analyzed by gas chromatography mass spectrometry (GC–MS) with a chiral column. Only one enantiomer (retention time: 6.5 min) was detected in the sample (Fig. 4a); its peak corresponded to the (*S*)-enantiomer peak of racemic linalool. Furthermore, the fragment ion mass spectrum of this product matched the spectral data of the linalool standard (Fig. 4b, c). These data reveal that 6×His-BLA-fusion did not affect the enantioselectivity of AaLINS.

### Fed-batch fermentation with IP04/pBLAAaLINS-ispA* strain

**Fig. 5:**
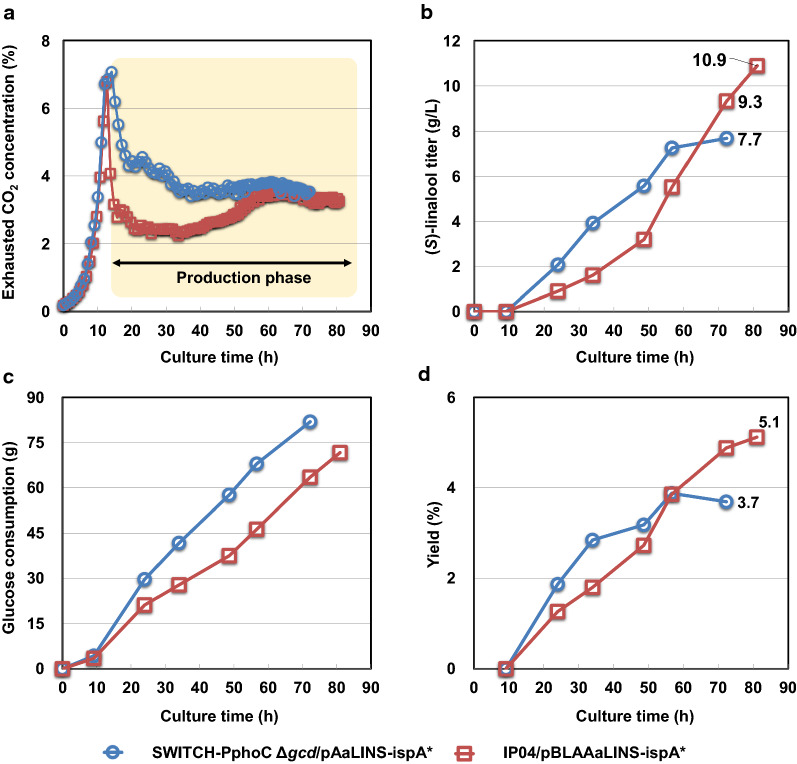
Typical time-course profiles of fed-batch fermentation of SWITCH-PphoC Δ*gcd*/pAaLINS-ispA* and IP04/pBLAAaLINS-ispA*. **a** CO_2_ concentration (%) in the exhausted gas; **b** (*S*)-linalool titer (g/L); **c** Consumed glucose amount (g); **d** Yield from glucose (%^−w/w^)

To investigate the (*S*)-linalool-producing ability of IP04S/pBLAAaLINS-ispA*, a fed-batch fermentation, which is relevant to industrial processes, was conducted in a 1-L scale fermenter; SWITCH-PphoC Δ*gcd*/pAaLINS-ispA* was also cultured as a control strain. One prominent strategy for high terpene production is to divide fermentation into a growth-phase and a subsequent production-phase (dual-phase fermentation), as this method can bypass the allocation of substrate between cell-growth and the target product and alleviate the effects of the accumulation of cytotoxic intermediates from the MVA pathway during the growth phase [28]. An external P_i_-dependent dual-phase fed-batch fermentation process was previously established for isoprene production using the SWITCH-PphoC strain [23]. The P_i_-starvation-inducible metabolic switch enables cells to grow efficiently under the P_i_-saturated phase and efficiently produce (*S*)-linalool under the subsequent P_i_-starved phase. Thus, a P_i_-starved fed-batch fermentation (1.8 g/L of KH_2_PO_4_) was conducted using the biphasic fermentation system. The culture temperature was optimized from the previous study [23] in which the culture temperature was set at 33 °C. The results are summarized in Fig. 5. As the optical density at 600 nm (OD_600_) could not represent the biomass concentration accurately owing to oil-in-water emulsion formation in biphasic fermentation [40], the profile of the CO_2_ concentration in the exhausted gas (ExCO_2_) was exploited as an index of total cell activity in the fermenter. The patterns of ExCO_2_ showed almost no difference between the two strains for up to 13 h of cultivation (Fig. 5a), which indicates that both strains grew with the same efficiency regardless of their genotypic differences. This result demonstrates that our P_i_-dependent dual-phase process contributed to overcoming both the competition for acetyl-CoA between cell-growth and (*S*)-linalool and intracellular accumulation of cytotoxic compounds in the growth phase. This feature of dual-phase fermentation also enables estimation of the approximate cell density when transiting to (*S*)-linalool production phase (P_i_-starved phase), despite the difficulty of monitoring the actual cell density with OD_600_ values in biphasic fermentation. The growth profile during the P_i_-saturated phase should be nearly the same regardless of the existence of IPM and genotypic differences. When the strain SWITCH-PphoC Δ*gcd*/pAaLINS-ispA* was cultured (1.8 g/L of KH_2_PO_4_) without IPM, its OD_600_ value at 13 h was 33. The ExCO_2_ of both strains declined at 13 h of cultivation (Fig. 5a), demonstrating that external P_i_-starvation started at this time. After entering the P_i_-starved phase, the ExCO_2_ profile of IP04/pBLAAaLINS-ispA* was lower than that of SWITCH-PphoC Δ*gcd*/pAaLINS-ispA* (Fig. 5a), indicating that IP04/pBLAAaLINS-ispA* redirected higher carbon flux from acetyl-CoA to the MVA pathway from the tricarboxylic acid (TCA) cycle, which generates CO_2_ and NADH for cell respiration, as compared to SWITCH-PphoC Δ*gcd*/pAaLINS-ispA*. SWITCH-PphoC Δ*gcd*/pAaLINS-ispA* accumulated higher levels of (*S*)-linalool than IP04/pBLAAaLINS-ispA* until approximately 60 h of cultivation (Fig. 5b). However, at 72 h of cultivation, despite less sugar consumption (63.5 g, Fig. 5c), IP04/pBLAAaLINS-ispA* produced larger amounts of (*S*)-linalool (9.3 g/L) than SWITCH-PphoC Δ*gcd*/pAaLINS-ispA*, which produced 7.7 g/L of (*S*)-linalool from 81.4 g of glucose (3.7% yield). A similar fermentation profile was observed in the other fed-batch fermentation (Additional file 1: Figure S4), though the culture temperature and initial KH_2_PO_4_ concentration (1.6 g/L) were different. Since the (*S*)-linalool titer of IP04/pBLAAaLINS-ispA* was still linearly increasing at 72 h of cultivation unlike SWITCH-PphoC Δ*gcd*/pAaLINS-ispA* (Fig. 5b), the culture time of IP04/pBLAAaLINS-ispA* was elongated to 81 h. As a result, IP04/pBLAAaLINS-ispA* produced a total of 10.9 g/L (final concentration) of (*S*)-linalool from 72.4 g of glucose (5.1% yield), whereas it accumulated 7.2 g/L of MVA in the aqueous culture medium as a main by-product.

## Discussion

Although advancements in the field of metabolic engineering have improved the production level of terpenes, linalool production has been confined to the mg/L-scale [12, 13, 15, 29–31]. However, our metabolically engineered *P. ananatis* strain IP04/pBLAAaLINS-ispA* produced enantiopure (*S*)-linalool with a 10.9 g/L titer under P_i_-dependent dual-phase fed-batch fermentation. This is the highest reported titer for microbial production of not only (*S*)-linalool but also all monoterpenes [10].

In this study, the use of *AaLINS* (codon-optimized for *Synechocystis*) unexpectedly led to higher AaLINS expression compared to the use of *AaLINS_pa* (codon-optimized for *P. ananatis*). When we previously evaluated 8 homologs of AaLINS, the codons of which were optimized for *P. ananatis* based on the OptimumGene algorithm [41], in SWITCH-PphoC Δ*gcd*, the linalool titers were less than 120 mg/L, although SWITCH-PphoC Δ*gcd*/pAaLINS-ispA* produced 1.6 g/L of (*S*)-linalool [26]. Further studies of the codon-optimization of linalool synthases are required. We also found that AaLINS was mainly expressed as insoluble form in *P. ananatis*. To improve AaLINS solubility, a solubility-tag fusion approach was applied to AaLINS. This approach is one of the commonly used methods for increasing the solubility of “difficult-to-express” heterologous proteins in bacterial cells. As a result of screening commonly used solubility-tags, 6×His-BLA was identified as a suitable one for AaLINS to increase its soluble production level; however, AaLINS aggregation could not be completely avoided by the N-terminal 6×His-BLA-fusion as observed in SDS–PAGE analysis, which demonstrates the positive effect of 6×His-BLA-fusion on solubility improvement is limited. Additionally, the mechanism of action underlying differences in solubility-tag efficacy has not been investigated. Furthermore, it has been revealed that 6×His-fusion may increase the expression level and activity, as seen in a previous study of a hyaluronidase production in a methylotrophic yeast [42].

Unlike SWITCH-PphoC Δ*gcd*/pAaLINS-ispA*, IP04/pAaLINS-ispA* was unable to completely consume the initial glucose in 48 h as well as IP04/pACYC177 in test-tube cultivation. IP04/pAaLINS-ispA* likely stopped consuming glucose after the start of P_i_ starvation because of over-accumulation of cytotoxic IPP/DMAPP, which led to a lower (*S*)-linalool titer. Compared to SWITCH-PphoC Δ*gcd*, IP04 is more likely to accumulate these cytotoxic intermediates intracellularly because of enhanced carbon flux to the MVA pathway when AaLINS activity was not high enough to avoid their excess accumulation. This issue was attenuated by increased intracellular AaLINS activity via 6×His-BLA-fusion. IP04/pBLAAaLINS-ispA* completely consumed the initial glucose and showed higher productivity compared to SWITCH-PphoC Δ*gcd*/pBLAAaLINS-ispA*.

Our P_i_-exploiting dual-phase fed-batch fermentation process has advantages for industrial-scale production, including simple operation to transit cells to the production phase, no requirement for exogenous inducers, and restricted respiratory activity, which is required for aerobic fermentation at industrial scale in order to fulfill technical constraints such as oxygen and heat transfer. Furthermore, we can aim to achieve the theoretical (*S*)-linalool yield from glucose in the production (P_i_-starved) phase. This is because ATP production and consumption can be stoichiometrically balanced from glucose to (*S*)-linalool via standard Embden-Meyerhof-Parnas (EMP) glycolysis and the MVA pathway [9, 43]. Four moles of NADPH, which are required to yield one mole of (*S*)-linalool from 6 acetyl-CoA via the MVA pathway, can be supplied via the EMP glycolysis by providing 12 NADH to yield 6 acetyl-CoA from 3 glucose as long as NAD(P) transhydrogenase (locus tag PAJ_1324 and 1325) is functional [9, 43]. To realize this, we aim to improve our P_i_-dependent metabolic switch to allow for dynamic metabolic control by developing conditional metabolic on/off systems [44] to shut off carbon flux to competing pathways such as TCA cycle and oxidative pentose-phosphate pathway, which is estimated to be upregulated by P_i_-starvation [45].

The yield (5.6%) in fed-batch fermentation was lower than that in the test-tube cultivation (7.9 ± 0.2%). However, the yield could be increased by elongating cultivation time (production-phase), as the cumulative yield continued to increase along with the culture time at the termination of production (Fig. 5d). Our next target is to further increase the (*S*)-linalool yield and titer; however, the upper limit of the titer is closely related to the properties and amount of in situ extraction organic solvent (IPM). Higher production of (*S*)-linalool by the *P. ananatis* strain leads to a higher (*S*)-linalool titer in the aqueous medium, according to the (*S*)-linalool distribution coefficient for IPM. Once more than 1 g/L of cytotoxic (*S*)-linalool accumulated in the aqueous phase, the growth and metabolic activity of *P. ananatis* were significantly reduced (Additional file 1: Figure S5), as reported for other Gram-negative bacteria [11, 46], and (*S*)-linalool production would be hampered. IPM has been used as an organic solvent in our studies [15, 26] because of its high biocompatibility with microbes and high distribution coefficient of monoterpenes [25]. However, IPM is not an economically viable solvent; thus, not only increasing the titer but also enhancing the resistance to monoterpene toxicity of *P. ananatis* [46, 47] to decrease the amount of IPM are necessary to reduce the production cost for industrial production.

IP04/pBLAAaLINS-ispA* accumulated 7.2 g/L of MVA into the medium at the end of fed-batch fermentation, which suggests that a pathway downstream of (*S*)-linalool biosynthesis, particularly AaLINS activity, may still be a potent bottleneck in this strain. Therefore, additional means may be required to increase intracellular (*S*)-linalool synthase activity. Lowering the culture temperature or co-expression of chaperones, which is known to improve heterologous protein solubility [48], should be considered; using protein engineering to screen mutant variants of AaLINS with improved performance is also an option [35, 36]. Another prominent option is using other linalool synthases that are known to produce only the (*S*)-enantiomer such as those from *Cinnamomum osmophloeum* [49] or *Malus domestica* [50] if their kinetic parameters and solubilities in bacteria are superior to those of AaLINS. Additionally, our platform strain and fermentation process can theoretically synthesize a multitude of different monoterpenes with almost the same productivity as (*S*)-linalool by changing only AaLINS to other mono-TPS, although it would depend on the solubility/stability and kinetic parameters of mono-TPS.

## Conclusions

We achieved a 10.9 g/L titer of (*S*)-linalool on the basis of SWITCH-PphoC Δ*gcd*/pAaLINS-ispA* via three main approaches: (1) improving intracellular activity of AaLINS, (2) increasing the precursor (GPP) supply, and (3) applying dual-phase fed-batch fermentation. Our results demonstrate that fermentative enantiopure “natural” (*S*)-linalool production with a metabolically engineered *P. ananatis* strain is a promising system that is environmentally-friendly and can be readily industrialized, although additional studies are needed to improve the economic viability of this process. Mass production of enantiopure (*S*)-linalool may contribute to accurate assessment of its biological properties, as most studies have been performed with (*R*)-linalool or linalool racemate [11].

## Methods

### Bacterial strains, plasmids, and growth conditions

The primary bacterial strains and plasmids used in this study are listed in Table 2. Other strains and plasmids used as materials for strain construction are listed in Additional file 1: Table S2. The primers used in this study are listed in Additional file 1: Table S3. *E. coli* strain JM109 (Takara Bio, Otsu, Japan) was primarily used for plasmid cloning and propagation. The DNA fragment was cloned into a linearized vector with an In-Fusion® HD cloning kit (Takara Bio). The plasmid was transformed into *P. ananatis* as previously reported [19]. Antibiotics were used to maintain plasmids or screen antibiotic-resistant transformants with the following concentrations: chloramphenicol (Cm: 60 mg/L), kanamycin (Km: 50 mg/L), and tetracycline (Tet: 10 mg/L).

**Table 2:** Bacterial strains and plasmids used in this study

Strain or plasmid	Description	Antibiotic resistance^a^	Source or reference
*Pantoea ananatis*			
SC17(0)	λ Red resistant strain	–	[19]
SWITCH-PphoC Δ*gcd*	SC17(0) Δ*ampC*::P_*tac*_-KDyI Δ*ampH*::P_*phoC*_*-mvaES* Δ*crtEXYIB-crtZ*::P_*tac*_-*mvk* Δ*gcd*	–	[26]
IP03	SWITCH-PphoC Δ*gcd* Δ*L-ldh*::P_*phoC*_-*mvaES*	–	This study
IP04	IP03 Δ*adhE*::P_*tac*_-φ10-*mvk*	–	This study
Plasmid			
pSol-AaLINS	pSol plasmid for expression of *AaLINS* under the control of a rhamnose-inducible promoter	Km	This study
pSol-HisAaLINS	pSol plasmid for expression of hexahistidine-tagged AaLINS	Km	This study
pSol-BLAAaLINS	pSol plasmid for expression of AaLINS fused with β-lactamase from *Chromohalobacter* sp. 560 joined to hexahistidine	Km	This study
pACYC177-P_*tac*_-*AaLINS*-ispA* (pAaLINS-ispA*)	pACYC177 derivative for expression of *AaLINS* and ispA* under the control of *tac* promoter	Km	[15]
pBLAAaLINS-ispA*	pACYC177 derivative for expression of the gene of AaLINS fused with β-lactamase from *Chromohalobacter* sp. 560 joined to hexahistidine and ispA* under the control of *tac* promoter	Km	This study

*Km* kanamycin

### Construction of plasmids for expressing solubility-tag fused AaLINS

An Expresso Solubility and Expression Screening System (Lucigen Corp., Middleton, WI, USA) was used to fuse each solubility-tag to AaLINS in *E. coli* strain E. cloni® 10G, according to the manufacturer’s protocol [39]. A DNA fragment of *AaLINS* was PCR-amplified from pAaLINS-ispA* using primers Lin-fw/Lin-rv and cloned into the linear pSol vectors [39]; the obtained plasmids are listed in Table 2 and Additional file 1: Table S2. A DNA fragment of *AaLINS* was PCR-amplified from pAaLINS-ispA* using primers P19/Lin-rv and then ligated to a vector fragment, which was PCR-amplified from pSol-BLAAaLINS using primers pSOL-fw/pSOL-rv, to yield pSol-AaLINS. A DNA fragment of the gene of 6×His-BLA fused AaLINS was PCR-amplified from pSol-BLAAaLINS using primers His-fw/LIS-rv and then ligated to a vector fragment, which was PCR-amplified from pAaLINS-ispA* using primers P-fw/P-rv to yield pBLAAaLINS-ispA*. The sequence data for the genes of AaLINS and IspA*, which were optimized based on the codon-preference of *Synechocystis*, are available in GenBank (GenBank accession numbers: LX078595.1 and LX078599.1).

### Construction of φ80-integrative conditional replication, integration, and modular (CRIM) plasmid, pAH162-P_*tac*_-φ10-*mvk*

The DNA fragment containing *mvk* from *M. paludicola* was PCR-amplified from pAH162-P_*tac*_-*mvk* [23] using primers P1/P2, and then cloned into a linearized vector that was PCR-amplified with primers P3/P4 from pIspSM [23] to yield pSTV28-P_*tac*_-φ10-*mvk*. The DNA fragment containing the P_*tac*_-φ10-*mvk* region was PCR-amplified from pSTV28-P_*tac*_-φ10-*mvk* using primers P5/P6, and then ligated to the *Pst*I/*Hind*III-digested pAH162-λ*attL*-Tc^R^-λ*attR* [51] and transformed into *E. coli* strain PIR2 (Thermo Fisher Scientific, Waltham, MA, USA).

### Construction of strains IP03 and IP04 using the Dual-In/Out strategy

Strains IP03 and IP04 were constructed from SWITCH-PphoC Δ*gcd* [26] by the Dual-In/Out strategy [20, 51]. The φ80*attL*-Km^R^-φ80*attR* DNA fragment flanked with 50-bp homologous to the *L-ldh* (locus tag PAJ_p0276) and the *adhE* (locus tag PAJ_1411) site was PCR-amplified from pMWattphi [51] using primers Ldh-F/Ldh-R and adhE-F/adhE-R, respectively. To create a φ80*attB* site in SC17(0), the PCR product was transformed by λRed-dependent homologous recombination [19]. After the removal of helper plasmid and Km^R^-marker gene [20], SC17(0) Δ*L-ldh*::φ80*attB* and SC17(0) Δ*adhE*::φ80*attB* were generated, respectively. To obtain strains SC17(0) Δ*L-ldh*::pAH162-P_*phoC*_-*mvaES* and SC17(0) Δ*adhE*::pAH162-P_*tac*_-φ10-*mvk*, the CRIM plasmid pAH162-P_*phoC*_-*mvaES* was integrated into SC17(0) Δ*L-ldh*::φ80*attB*, and pAH162-P_*tac*_-φ10-*mvk* was integrated into SC17(0) Δ*adhE*::φ80*attB* by φ80-dependent recombination with pAH123-cat [20]. The genomic DNA of SC17(0) Δ*L-ldh*::pAH162-P_*phoC*_-*mvaES* was purified, fragmented, and then electroporated into SWITCH-PphoC Δ*gcd* to transfer the chromosomal modification (Δ*L-ldh*::pAH162-P_*phoC*_-*mvaES* [Tet^R^]) as previously reported [19]. The marker- and plasmid-less strain, IP03, was obtained by λ integrase and excisionase-dependent marker excision with pMW-Int/Xis-cat [19]. Following the same procedure, strain IP04 (SWITCH-PphoC Δ*gcd* Δ*L-ldh*::P_*phoC*_-*mvaES* Δ*adhE*::P_*tac*_-φ10-*mvk*) was generated from IP03 with the genomic DNA of SC17(0) Δ*adhE*::pAH162-P_*tac*_-φ10-*mvk*.

### Preparation of the bacterial lysate

The SC17(0) strains harboring each pSol plasmid for expressing each AaLINS variant or AaLINS were grown on Luria–Bertani (LB)-agar for 16 h at 34 °C. A single colony was inoculated into 3 mL of LB liquid medium containing 0.4 g/L glucose and 4 g/L of rhamnose. After cultivation at 30 °C for 21 h on a reciprocal shaker at 120 rpm (OD_600_ was approximately 6), the harvested cells were washed twice with extraction buffer (50 mM MOPS [pH 7.0], 10 mM MgSO_4_·5H_2_O, 10% [v/v] glycerol, and 1 mM dithiothreitol), and re-suspended in the same buffer. The cells were disrupted by sonication at 4 °C to obtain the crude homogenate including soluble and insoluble proteins. The crude homogenate was centrifuged (21,600 × *g*, 10 min, 4 °C) to obtain the supernatant or soluble protein fraction.

### SDS–PAGE analysis

Protein concentration was quantified with a Pierce BCA protein assay kit (Thermo Fisher Scientific). The soluble protein fraction containing 10 µg of protein was subjected to SDS–PAGE, and the crude homogenate was applied with the same volume (µL) of the corresponding soluble protein fraction. The samples were reduced at 70 °C for 10 min with NuPAGE Sample Reducing Agent (Thermo Fisher Scientific). Proteins were separated on a NuPAGE 4–12% Bis–Tris protein gel (Thermo Fisher Scientific) with MOPS SDS Running Buffer (Thermo Fisher Scientific) at 200 V for 90 min, and then stained with InVision His-Tag In-Gel Stain (Thermo Fisher Scientific) to visualize the His-tagged fusion proteins with an anti-polyhistidine label (nickel-nitrilotriacetic acid), according to the manufacturer’s protocol. Fluorescence images were obtained at an excitation wavelength of 520 nm. The gel was re-stained with CBB. An XL-Ladder Broad (intégrale Co., Ltd, Tokyo, Japan) was used as the molecular weight marker.

### Single-vial biotransformation assay

The protein concentration of the soluble protein fraction was diluted to 300 mg/L in a final volume of 1 mL extraction buffer, which was previously supplemented with 15 µM (final concentration) GPP lithium salt (Sigma-Aldrich, St. Louis, MO, USA), in a 22-mL Crimp Top vial (PerkinElmer, Waltham, MA, USA). For the assay with the crude homogenate, the same volume (µL) of the corresponding soluble protein fraction was applied in 1 mL extraction buffer with GPP. The vials were tightly capped with a 20 mm Crimp Top Aluminum Silver Cap with PTFE/Butyl Septa (PerkinElmer) and incubated at 30 °C for 26 h on a reciprocal shaker (120 rpm). Two hundred microliters of IPM were injected into the vial, which was thereafter vigorously shaken to extract (*S*)-linalool from the reaction buffer. The IPM layer was diluted by fivefold with ethanol, which was used to quantify (*S*)-linalool using a gas chromatography flame-ionization detector (GC–FID) as described below. Triplicate reactions were performed with bacterial lysates from three independent transformant colonies.

### (*S*)-linalool production in test tubes

Cultivation was conducted essentially as previously reported [26]. The concentrations of glucose and KH_2_PO_4_ in the medium were set at 60 and 0.5 g/L, respectively. The test tubes were shaken at 30 °C for 48 h in a reciprocal shaker (120 rpm). At least triplicate cultivations with independent transformant colonies were evaluated.

### Fed-batch fermentation in 1-L fermenter

The cells were incubated at 34 °C for 16 h on an LB-agar plate including Km (dish diameter: 88 mm). Whole cells on a plate were harvested with a 10-µL inoculation loop and then transferred into 270 mL of medium (44 g/L glucose, 1.1 g/L MgSO_4_·7H_2_O, 2.2 g/L Bacto yeast extract [BD Biosciences, Franklin Lakes, NJ, USA], 1.8 g/L KH_2_PO_4_, 1.1 g/L (NH_4_)_2_SO_4_, 1.1 g/L trisodium citrate, 11 mg/L MnSO_4_·5H_2_O, 11 mg/L FeSO_4_·7H_2_O, 0.11 mL/L antifoam reagent GD-113 K [NOF Corporation, Tokyo, Japan] and Km) overlaid with 30 mL of IPM in a 1-L fermenter. The glucose solution (700 g/L) containing 0.7 mL/L of GD-113 K was continuously fed from 9 h to the end of cultivation to maintain the glucose concentration at more than 5 g/L. The feeding rate was at approximately 1.5 mL/h for IP04/pBLAAaLINS-ispA* and at approximately 2.0 mL/h for SWITCH-PphoC Δ*gcd*/pAaLINS-ispA*. Fermentation was aerobically conducted with 300 mL/min aeration; the culture temperature was set at 34 °C for 15 h, and then shifted to 30 °C and kept at 30 °C until the end of cultivation; the culture pH was maintained at 6.8 with ammonia gas. The oxygen and CO_2_ concentrations in the exhausted-gas were measured every hour with an exhaust oxygen CO_2_ meter Model EX-1562–1 (Able & Biott Co., Tokyo, Japan).

### Analysis of metabolites

The OD_600_ was measured using a spectrometer (U-2900; Hitachi, Tokyo, Japan). The (*S*)-linalool concentration was quantified as follows. After vigorously vortexing the culture samples (mixture of cells, medium, and IPM), 100 μL of the aliquot was added to 900 μL of ethanol. These diluted samples were centrifuged (21,600 × *g*, 5 min, 4 °C). The supernatants were used for (*S*)-linalool quantification with a GC-2025AF (Shimadzu, Kyoto, Japan) equipped with DB-5 capillary column (diameter, 0.25 mm; length, 30 m; thickness, 0.25 µm) (Agilent Technologies, Santa Clara, CA, USA) and FID as previously reported [15, 26]. The concentration of (*S*)-linalool produced was quantified using a standard curve. (*S*)-linalool concentration is represented, being assumed to completely exist in aqueous culture. After fractionizing the biphasic fermentation broth into a cell pellet, aqueous supernatant, and IPM fraction by centrifugation (21,600 × *g*, 10 min, 4 °C), glucose and MVA concentrations in the aqueous supernatant were quantified as previously reported [23]. The IPM fraction was used to identify the product, and the type of enantiomer was determined by GC–MS (Agilent 7890A GC and 5975C MSD, Agilent Technologies) equipped with a chiral GC capillary column Rt-bDEXsm (RESTEK Corporation, Bellefonte, PA, USA) (diameter, 0.25 mm; length, 30 m; thickness, 0.25 µm) using helium as the carrier gas. The injector temperature was maintained at 230 °C. The GC oven temperature gradient was as follows: 115 °C hold for 10 min, increased to 225 °C (10 °C/min), and held for 9 min. (*R*)- and (*S*)-linalool racemic reagent (Fujifilm Wako Pure Chemical, Osaka, Japan, catalog number: 126–00,993) and (*R*)-linalool reagent (Sigma-Aldrich, catalog number: 62139-25ML) were used to confirm the retention times of the (*R*)- and (*S*)-enantiomers.

## Supplementary Information

**Additional file 1: Figure S1.** Nucleotide sequences of *AaLINS_pa* and ispA*_pa. **Figure S2.** Construction of plasmids for over-expression of both AaLINS_pa and IspA*_pa. **Figure S3.** SDS–PAGE gel illustrating total, soluble, and insoluble expression of AaLINS and AaLINS_pa. **Figure S4.** Typical time-course profiles of fed-batch fermentation (1.6 g/L of KH_2_PO_4_) of SWITCH-PphoC Δ*gcd*/pAaLINS-ispA* and IP04/pBLAAaLINS-ispA*. **Figure S5.** Growth inhibition of *P. ananatis* SC17 strain by exogenous linalool. **Table S1.** (*S*)-Linalool production in test-tube cultivation. **Table S2.** Bacterial strains and plasmids used for strain construction. **Table S3.** Primers used in this study.

## Data Availability

All data generated or analyzed during this study are included in this published article and its additional file.

## Abbreviations

AaLINS: (*S*)-linalool synthase from *Actinida arguta*
AFV: AFV1-99 protein from Acidianus filamentous virus 1
BLA: Halophilic β-lactamase from *Chromohalobacter* sp. 560
CBB: Coomassie Brilliant Blue
Cm: Chloramphenicol
CoA: Coenzyme A
CRIM: Conditional replication, integration, and modular
DMAPP: Dimethylallyl pyrophosphate
EMP: Embden–Meyerhof–Parnas
ExCO_2_: Carbon dioxide in exhausted gas
FID: Flame-ionization detector
6×His: Hexahistidine
GC: Gas chromatography
GPP: Geranyl pyrophosphate
Int: Integrase
IPM: Isopropyl myristate
IPP: Isopentenyl pyrophosphate
IspA*: S80F mutant of farnesyl pyrophosphate synthase from *E. coli*
Km: Kanamycin
LB: Luria–Bertani
MBP: Maltose binding protein
MOPS: 3-(N-morpholino)propanesulfonic acid
MS: Mass spectrometry
MVA: Mevalonate
OD: Optical density
PCR: Polymerase chain reaction
P_i_: Inorganic phosphate
SDS–PAGE: Sodium dodecyl sulfate–polyacrylamide gel electrophoresis
SlyD: FKBP-type peptidyl-prolyl *cis–trans* isomerase
SUMO: Small ubiquitin-like modifier
TCA: Tricarboxylic acid
Tet: Tetracycline
TPS: Terpene synthase
Tsf: *E. coli* Elongation factor
Xis: Excisionase
