# Supplementary material for: Fermentative production of enantiopure (S)-linalool using a metabolically engineered Pantoea ananatis
Source: Microb Cell Fact. 2021 Mar 2;20:54. doi: 10.1186/s12934-021-01543-0 (PMC7923825; doi:10.1186/s12934-021-01543-0)
Supplement: Supplementary file 1 — Additional file 1: Figure S1. Nucleotide sequences of AaLINS_pa and ispA*_pa. Figure S2. Construction of plasmids for over-expression of both AaLINS_pa and IspA*_pa. Figure S3. SDS–PAGE gel illustrating total, soluble, and insoluble expression of AaLINS and AaLINS_pa. Figure S4. Typical time-course profiles of fed-batch fermentation (1.6 g/L of KH2PO4) of SWITCH-PphoC Δgcd/pAaLINS-ispA* and IP04/pBLAAaLINS-ispA*. Figure S5. Growth inhibition of P. ananatis SC17 strain by exogenous linalool. Table S1. (S)-Linalool production in test-tube cultivation. Table S2. Bacterial strains and plasmids used for strain construction. Table S3. Primers used in this study. [file 12934_2021_1543_MOESM1_ESM.docx]

**Additional File 1**

**Additional Figures and Tables**

**Fermentative production of enantiopure (*S*)-linalool using a metabolically engineered *Pantoea ananatis***

Nobuhisa Nitta,^#^ Yoshinori Tajima, Yoko Yamamoto, Mika Moriya, Akiko Matsudaira, Yasushi Hoshino, Yousuke Nishio, Yoshihiro Usuda

Research Institute for Bioscience Products & Fine Chemicals, Ajinomoto Co., Inc., Kawasaki, Japan

^#^Address correspondence to Nobuhisa Nitta, [nobuhisa_nitta@ajinomoto.com](mailto:nobuhisa_nitta@ajinomoto.com).

**Contents**

**Figure S1.** Nucleotide sequences of *AaLINS_pa* and *ispA*_pa*.

**Figure S2.** Construction of plasmids for over-expression of both AaLINS_pa and IspA*_pa.

**Figure S3.** SDS–PAGE gel illustrating total, soluble, and insoluble expression of AaLINS and AaLINS_pa.

**Figure S4.** Typical time-course profiles of fed-batch fermentation (1.6 g/L of KH_2_PO_4_) of SWITCH-PphoC Δ*gcd*/pAaLINS-ispA* and IP04/pBLAAaLINS-ispA*.

**Figure S5.** Growth inhibition of *P. ananatis* SC17 strain by exogenous linalool.

**Table S1.** (*S*)-Linalool production in test-tube cultivation

**Table S2.** Bacterial strains and plasmids used for strain construction

**Table S3.** Primers used in this study

**Figure S1.** Nucleotide sequences of *AaLINS_pa* and *ispA*_pa*. *AaLINS_pa*, (*S*)-linalool synthase gene which was codon-optimized to match the codon-preference of *Pantoea ananatis*; *ispA*_pa*, gene of the S80F mutant of farnesyl pyrophosphate synthase which was codon-optimized to match the codon-preference of *P. ananatis*.

**>*AaLINS_pa* gene**

ATGAGCACGGCGGTTCCCAGCATGCCCACCACCCAGAAATGGAGCATCACGGAGGACCTGGCGTTCATCAGCAATCCCAGCAAACAGCATAACCACCAGACCGGCTACCGCATTTTTAGCGATGAATTCTACCTGAAACATGAAAACAAACTGAAAGATGTGCGTCGCGCCCTGCGCGAAGTGGAAGAAACCCCGCTGGAAGGCCTGGTGATGATCGATACCCTGCAGCGCCTGGGCATTGATTACCACTTTCAGGGCGAAATCGGCGCGCTGCTGCAGAAACAGCAGCGCATTAGCACCTGCGATTATCCGGAACATGACCTGTTTGAAGTGAGCACGCGTTTTCGTCTGCTGCGTCAGGAAGGCCACAATGTGCCGGCCGATGTGTTTAACAACTTTCGCGATAAAGAAGGCCGCTTTAAAAGCGAACTGAGCCGCGATATCCGCGGCCTGATGAGCCTGTACGAAGCCAGCCAGCTGAGCATCCAGGGCGAAGATATTCTGGATCAGGCCGCGGATTTTAGCAGCCAGCTGCTGAGCGGTTGGGCCACCAACCTGGATCATCATCAGGCCCGTCTGGTGCGTAATGCCCTGACCCATCCGTACCACAAAAGCCTGGCCACCTTTATGGCGCGCAACTTTAACTATGATTGCAAAGGCCAGAACGGCTGGGTGAACAACCTGCAGGAACTGGCCAAAATGGATCTGACCATGGTGCAGAGCATGCATCAGAAAGAAGTGCTGCAGGTGTCTCAGTGGTGGAAAGGTCGCGGCCTGGCGAACGAACTGAAACTGGTGCGCAACCAGCCGCTGAAATGGTATATGTGGCCGATGGCGGCCCTGACGGATCCGCGCTTTAGCGAAGAACGCGTGGAACTGACCAAACCGATCAGCTTCATCTACATCATCGATGATATCTTCGATGTGTACGGCACCCTGGAAGAACTGACCCTGTTTACCGATGCCGTGAACCGCTGGGAACTGACCGCGGTGGAACAGCTGCCGGATTACATGAAAATTTGCTTTAAAGCCCTGTACGATATCACCAACGAAATCGCGTACAAAATCTACAAAAAACACGGCCGCAACCCGATCGATAGCCTGCGTCGCACCTGGGCCAGCCTGTGCAACGCGTTTCTGGAAGAAGCCAAATGGTTTGCGAGCGGCAACCTGCCGAAAGCCGAAGAATACCTGAAAAACGGCATTATCAGCAGCGGCATGCATGTGGTGACCGTGCACATGTTTTTCCTGCTGGGCGGCTGCTTTACCGAAGAAAGCGTGAACCTGGTGGATGAACATGCCGGCATTACCAGCAGCATTGCGACCATCCTGCGCCTGAGCGATGATCTGGGCAGCGCGAAAGATGAAGATCAGGATGGCTATGATGGCAGCTACCTGGAATGCTATCTGAAAGATCACAAAGGCAGCAGCGTGGAAAACGCCCGCGAAGAAGTGATTCGCATGATCAGCGATGCGTGGAAACGCCTGAACGAAGAATGCCTGTTTCCGAACCCGTTTAGCGCCACCTTTCGCAAAGGCAGCCTGAACATCGCGCGCATGGTGCCGCTGATGTATAGCTACGATGATAACCACAACCTGCCCATTCTGGAGGAACACATGAAAACGATGCTGTATGATAGCAGCAGCTAA

> ***ispA*_pa* gene**

ATGGACTTCCCCCAGCAGCTGGAGGCGTGCGTCAAACAGGCCAATCAGGCCCTGAGCCGTTTCATCGCGCCCCTGCCCTTTCAGAATACCCCGGTGGTGGAAACCATGCAGTATGGTGCCCTGCTGGGCGGCAAACGTCTGCGTCCGTTTCTGGTGTACGCGACCGGCCATATGTTTGGCGTGAGCACGAATACCCTGGATGCCCCGGCGGCCGCCGTGGAATGCATTCATGCCTATTTTCTGATCCACGATGATCTGCCGGCGATGGATGATGATGATCTGCGCCGCGGCCTGCCGACCTGCCATGTGAAATTTGGCGAAGCGAACGCCATTCTGGCCGGTGATGCCCTGCAGACCCTGGCCTTTAGCATCCTGAGCGATGCCGATATGCCGGAAGTGAGCGATCGCGATCGCATTAGCATGATCAGCGAACTGGCCAGCGCCAGCGGTATTGCGGGCATGTGTGGTGGCCAGGCCCTGGATCTGGATGCCGAAGGCAAACATGTGCCGCTGGATGCCCTGGAACGCATTCATCGCCACAAAACGGGTGCCCTGATCCGTGCCGCGGTGCGTCTGGGCGCGCTGAGCGCCGGCGATAAAGGCCGTCGCGCCCTGCCGGTGCTGGATAAATATGCGGAAAGCATTGGCCTGGCCTTTCAGGTGCAGGATGATATCCTGGATGTGGTGGGCGATACGGCCACGCTGGGTAAACGTCAGGGTGCGGATCAGCAGCTGGGCAAAAGCACGTATCCGGCCCTGCTGGGTCTGGAACAGGCCCGCAAGAAAGCCCGCGATCTGATCGATGATGCGCGCCAGAGCCTGAAACAGCTGGCCGAACAGAGCCTGGACACGAGCGCCCTGGAAGCCCTGGCCGACTACATCATTCAGCGTAACAAATAA

**Figure S2.** Construction of plasmids for over-expression of both AaLINS_pa and IspA*_pa.

The nucleotide sequences of *AaLINS_pa* and *ispA*_pa* were optimized to match the codon-preference of *P. ananatis* by GenScript (Piscataway, NJ, USA). The nucleotide sequence including *tac* promoter, ribosomal binding site sequence (5'-CCCTGTTGACAATTAATCATCGGCTCGTATAATGTGTGGAATCGTGAGCGGATAACAATTTCACACAAGGAGACTGCC-3') and the DNA sequence of the *AaLINS_pa* (Figure S1) was chemically synthesized and cloned into pUC57 (GenScript) to yield pUC57-AaLINS_pa. The DNA sequence of the *ispA*_pa* (Figure S1) was also cloned into pUC57-Kan plasmid to yield pUC57-ispA*_pa (GenScript). A gene fragment including the *AaLINS_pa* was PCR-amplified with primers Ls1/P16 from pUC57-AaLINS_pa, and the gene fragment of the *ispA**_*pa* was PCR-amplified with primers P17/P18 from pUC57-ispA*_pa; the two fragments were inserted into *Pst*I/*Sca*I-digested pACYC177 (Nippon Gene, Toyama, Japan) using an In-Fusion HD cloning kit and transformed into *E. coli* JM109 (Takara Bio) to yield pAaLINS_pa-ispA*_pa. An Expresso Solubility and Expression Screening System (Lucigen) was used for fusing a halophilic β-lactamase from *Chromohalobacter* sp. 560 or hexahistidine-tag to AaLINS_pa with E. cloni^®^ 10G, according to the manufacturer’s protocol. A DNA fragment including both *AaLINS_pa* and *ispA**_*pa* genes was PCR-amplified from pAaLINS_pa-ispA*_pa using primers P11/P12 and was cloned into the linearized pSol Bla and pSol His Control to yield pSol-BLAAaLINS_pa-ispA*_pa and pSol-HisAaLINS_pa-ispA*_pa, respectively. A DNA fragment including the genes of 6*×*His-BLA-fused AaLINS_pa and IspA*_pa was PCR-amplified from pSol-BLAAaLINS_pa-ispA*_pa using primers His-fw/P15, and a DNA fragment including the genes of 6*×*His-tagged *AaLINS_pa* and IspA*_pa was PCR-amplified from pSol-HisAaLINS_pa-ispA*_pa using the same primers. Each PCR amplicon was ligated to a vector fragment, which was PCR-amplified from pAaLINS-ispA* using primers P13/P14, and transformed into JM109 to yield pBLAAaLINS_pa-ispA*_pa and pHisAaLINS_pa-ispA*_pa, respectively.

**
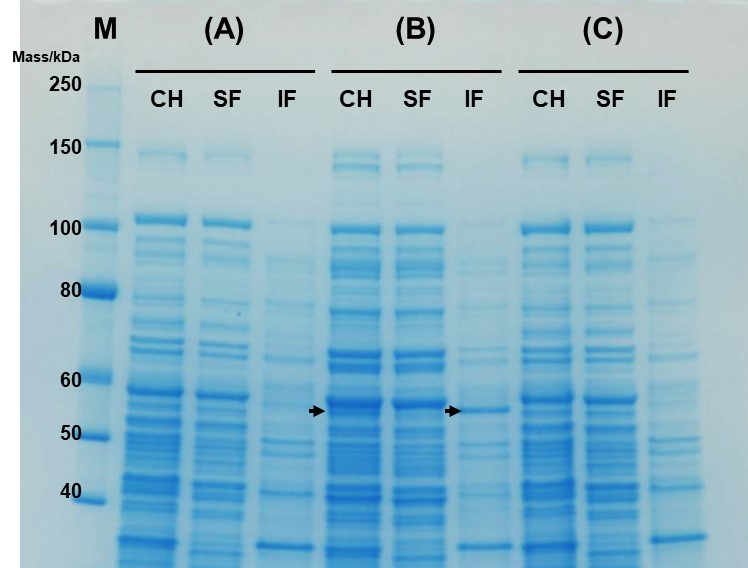
**

**Figure S3.** SDS–PAGE gel illustrating total, soluble, and insoluble expression of AaLINS and AaLINS_pa. Samples were prepared from SWITCH-PphoC Δ*gcd* strain harboring pACYC177 (A), pAaLINS-ispA* (B), and pAaLINS_pa-ispA*_pa (C). CH, SF, IF, and M denote crude homogenate, soluble fraction, insoluble fraction, and protein standard, respectively. A single colony of each strain on LB-agar containing 50 mg/L of kanamycin (Km) was inoculated into 3 mL of LB liquid medium in the presence of Km. Cultivation was conducted at 30°C for 21 h on a reciprocal shaker at 120 rpm. After 3 h of cultivation, 1 mM of isopropyl-β-D-thiogalactopyranoside was added. The harvested cells were washed twice and re-suspended in 400 µL of the extraction buffer. The cells were disrupted by sonication at 4°C. Two hundred µL of crude homogenate was centrifuged (21,600*×g*, 10 min, 4°C) to obtain the soluble protein fraction. The insoluble protein pellets were washed with the extraction buffer twice and resuspended in 200 µL of the extraction buffer containing 0.2 g/L sodium dodecyl sulfate to solubilize insoluble protein pellets (insoluble protein fraction). The soluble protein fraction containing 10 µg of protein was subjected to an SDS–PAGE gel, and both the insoluble protein fraction and the crude homogenate were applied with the same volume (µL) of the corresponding soluble protein fraction.


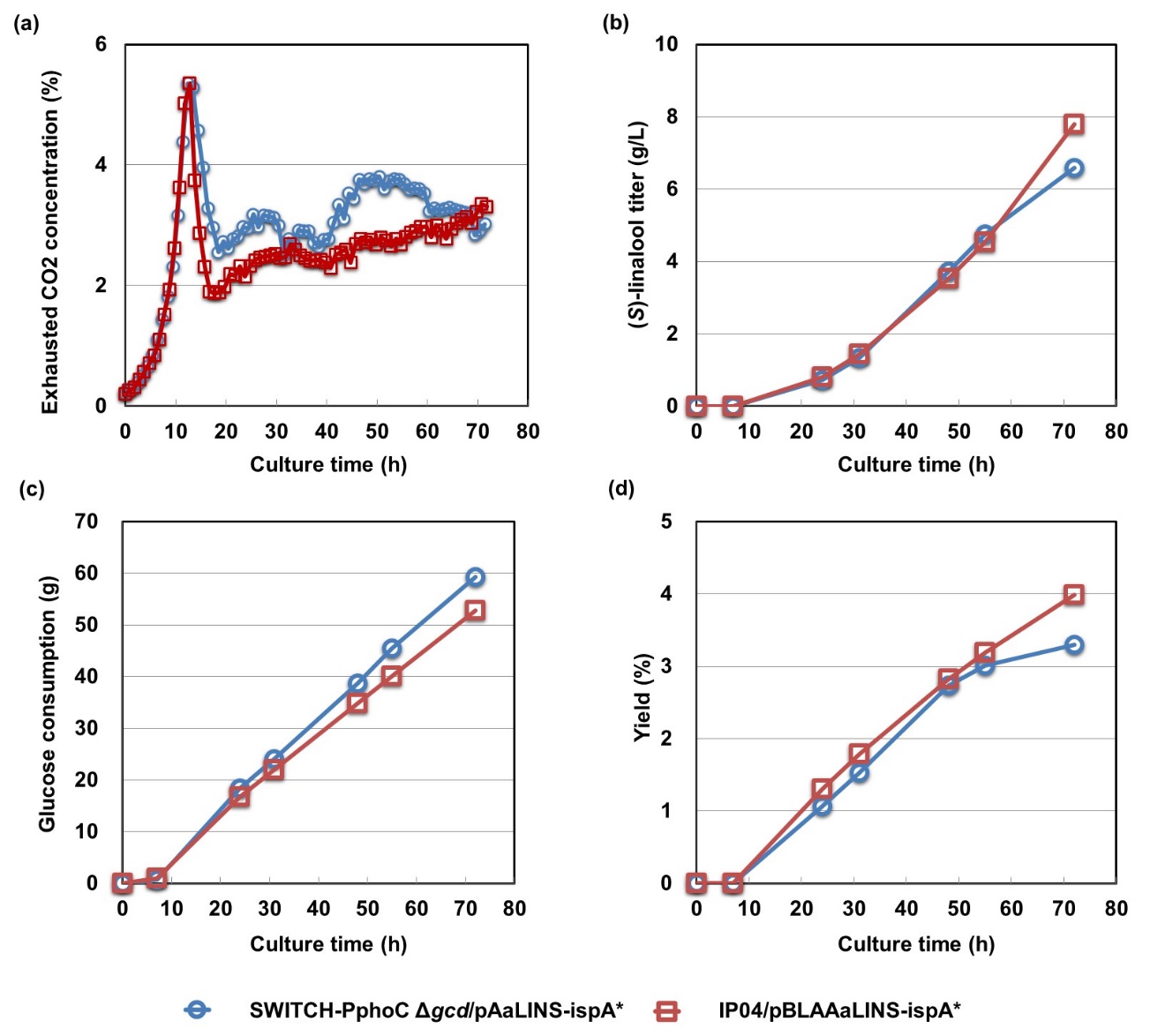


**Figure S4.** Typical time-course profiles of fed-batch fermentation (1.6 g/L of KH_2_PO_4_) of SWITCH-PphoC Δ*gcd*/pAaLINS-ispA* and IP04/pBLAAaLINS-ispA*. (a) CO_2_ concentration (%) in the exhausted gas; (b) (*S*)-linalool titer (g/L); (c) Consumed glucose amount (g); (d) Yield from glucose (%^-w/w^). The culture temperature was set at 30°C for 15 h, then shifted to 28°C and kept at 28°C until the end of cultivation (72 h).


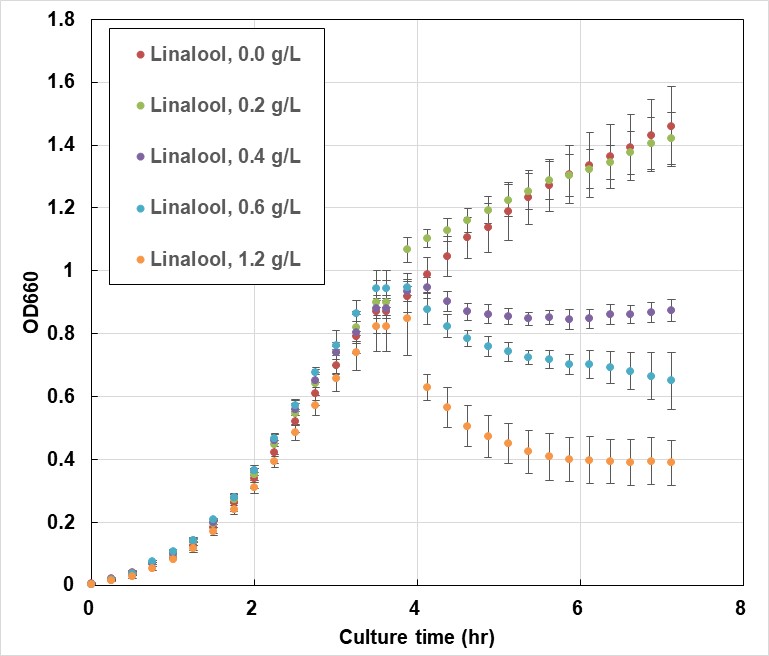


**Figure S5.** Growth inhibition of *P. ananatis* SC17 strain by exogenous linalool.

The harvested *P. ananatis* SC17 cells from LB-agar containing Km were inoculated into 4 mL of a modified M9 minimal medium (12.8 g/L Na_2_HPO_4_·7H_2_O, 3 g/L KH_2_PO_4_, 0.5 g/L NaCl, 2 g/L NH_4_Cl, 0.5 g/L MgSO_4_·7H_2_O, and 0.01 g/L CaCl_2_) supplemented with 4 g/L of glucose and 50 mg/L of Km; the initial bacterial turbidity (the optical density at 660 nm [OD_660_]) was adjusted to 0.002. To investigate the effects of exogenous linalool on the cell growth, SC17 strain was grown at 34°C at a shaking speed of 70 rpm. When the OD_660_ reached approximately 1.0, linalool racemic reagent (Fujifilm Wako Pure Chemical, Osaka, Japan) diluted in ethanol was added to culture to final concentrations of 1.2, 0.6, 0.4, 0.2 and 0 g/L, respectively. The OD_660_ values of the growing cultures were measured every 15 min with a TVS062CA Bio-Photorecorder (Advantec MFS Inc., Tokyo, Japan). An error bar shows a standard deviation deduced from three independent experiments.

**Table S1.** (*S*)-linalool production in test-tube cultivation

| Host strain/plasmid | (*S*)-linalool titer^a^  (g/L) | Glucose consumption^a^  (g/L) | Yield^b^  (%) | Mevalonate titer^a^  (g/L) |
| --- | --- | --- | --- | --- |
| SWITCH-PphoC Δ*gcd*/pAaLINS_pa-ispA*_pa | (13 ± 1.2) × 10^−3^ | 40 ± 0.6 | (3.2 ± 0.2) × 10^−2^ | 1.9 ± 0.0 |
| SWITCH-PphoC Δ*gcd*/pHisAaLINS_pa-ispA*_pa | (306 ± 49) × 10^−3^ | 41 ± 0.5 | (8.2 ± 0.1) × 10^−1^ | 1.3 ± 0.0 |
| SWITCH-PphoC Δ*gcd*/pBLAAaLINS_pa-ispA*_pa | 2.2 ± 0.2 | 60 ± 0.0 | 3.7 ± 0.2 | ND^c^ |

All strains were cultivated for 48 h under biphasic fermentation using isopropyl myristate. Data are expressed as mean ± SD of three independent experiments.

^a^Glucose, mevalonate, and (*S*)-linalool concentrations are represented by dividing the total amounts by the volume of aqueous culture.

^b^Yield was calculated as grams of product per grams of consumed glucose and is expressed as a percentage. Carbon sources contained in 2 g/L of Bacto yeast extract was not considered in this calculation.

^c^ND, Not detected.

**Table S2.** Bacterial strains and plasmids used for strain construction

| Strain or plasmid | Description | Antibiotic resistance^a^ | Source or reference |
| --- | --- | --- | --- |
| Strain |  |  |  |
| *Escherichia coli* |  |  |  |
| JM109 | Competent cells for plasmid cloning | − | Takara Bio |
| PIR2 | Competent cells for plasmid cloning | − | Invitrogen |
| E. cloni^®^ 10G | *F^−^ mcrA* Δ*(mrr-hsdRMS-mcrBC) endA1 recA1* φ*80dlacZ*Δ*M15* Δ*lac×74 araD139* Δ*(ara,leu)7697 galU galK rpsL nupGλ^−^ tonA* | − | Lucigen  [39] |
| *Pantoea ananatis* |  |  |  |
| SC17 | A low-mucus-producing mutant derived from wild-type AJ13355 | − | NITE^b^ |
| SC17(0) Δ*L-ldh*::φ80*attB* | SC17(0) Δ*L-ldh*::φ80*attB* | − | This study |
| SC17(0) Δ*adhE*::φ80*attB* | SC17(0) Δ*adhE*::φ80*attB* | − | This study |
| SC17(0) Δ*L-ldh*::pAH162-P*_phoC_*-*mvaES* | SC17(0) Δ*L-ldh*::pAH162-P*_phoC_*-*mvaES* | Tet | This study |
| SC17(0) Δ*adhE*::pAH162-P*_tac_*-φ10-*mvk* | SC17(0) Δ*adhE*::pAH162-P*_tac_*-φ10-*mvk* | Tet | This study |
| Plasmid |  |  |  |
| pSol-AFVAaLINS | pSol AFV derivative for expression of AaLINS fused with the AFV1–99 protein from Acidianus filamentous virus 1 and a rhamnose-inducible promoter | Km | This study |
| pSol-MBPAaLINS | pSol MBP derivative for expression of AaLINS fused with the Maltose-Binding Protein and a rhamnose-inducible promoter | Km | This study |
| pSol-SlyDAaLINS | pSol SlyD derivative for expression of AaLINS fused with the FKBP-type peptidyl-prolyl *cis-trans* isomerase-tag and a rhamnose-inducible promoter | Km | This study |
| pSol-SUMOAaLINS | pSol SUMO derivative for expression of AaLINS fused with the Small Ubiquitin-like Modifier and a rhamnose-inducible promoter | Km | This study |
| pSol-TsfAaLINS | pSol Tsf derivative for expression of AaLINS fused with the *E. coli* elongation factor and a rhamnose-inducible promoter | Km | This study |
| pAH162-P*_phoC_*-*mvaES* | CRIM plasmid^c^ | Tet | [23] |
| pAH162-P*_tac_*-*mvk* | CRIM plasmid | Tet | [23] |
| pAH162-P*_tac_*-φ10-*mvk* | Derivative of pAH162-P*_tac_*-*mvk* | Tet | This study |
| pIspSM | Plasmid for expression of an isoprene synthase from *Mucuna pururiens* | Cm | [23] |
| pSTV28-P*_tac_*-φ10-*mvk* | Derivative of pIspSM containing the region of P*_tac_*-φ10-*mvk* | Cm | This study |
| pAH129-cat | φ80Int/Xis expression plasmid^d^ | Cm | [20] |
| pAH123-cat | φ80Int expression plasmid | Cm | [20] |
| pAH162-λ*attL*-Tc^R^-λ*attR* | CRIM plasmid | Tet | [51] |
| pMWattphi | φ80*attL*-Km^R^-φ80*attR* cassette donor | Amp, Km | [51] |
| pRSFredTER | λ Red proteins expression plasmid | Cm | [19] |
| pMW-λInt/Xis-cat | λ Xis/Int expression plasmid | Cm | [19] |
| pUC57 | Plasmid vector with a replication origin of pMB1 | Amp | GenScript |
| pUC57-Kan | Plasmid vector with a replication origin of pMB1 | Km | GenScript |
| pUC57-AaLINS_pa | pUC57 derivative containing the sequence of *AaLINS_pa* | Amp | This study |
| pUC57-ispA*_pa | pUC57-Kan derivative containing the sequence of *ispA*_pa* | Km | This study |
| pSol-BLAAaLINS_pa-ispA*_pa | pSol Bla derivative for expression of AaLINS_pa fused with β-lactamase from *Chromohalobacter* sp. 560 and IspA*_pa | Km | This study |
| pSol-HisAaLINS_pa-ispA*_pa | pSol His Control derivative for expression of hexahistidine-tagged AaLINS_pa and IspA*_pa | Km | This study |
| pACYC177 | Plasmid vector with a replication origin of p15A | Amp, Km | Nippon Gene |
| pAaLINS_pa-ispA*_pa | pACYC177 derivative for expression of *AaLINS_pa* and *ispA*_pa* under the control of *tac* promoter | Km | This study |
| pHisAaLINS_pa-ispA*_pa | pACYC177 derivative for expression of hexahistidine-tagged AaLINS_pa and IspA*_pa | Km | This study |
| pBLAAaLINS_pa-ispA*_pa | pACYC177 derivative for expression of AaLINS_pa fused with β-lactamase from *Chromohalobacter* sp. 560 and IspA*_pa | Km | This study |

^a^Amp, ampicillin; Cm, chloramphenicol; Km, kanamycin; Tet, tetracycline.

^b^National Institute of Technology and Education.

^c^CRIM plasmid, conditional replication, integration, and modular plasmid.

^d^Int, gene encoding integrase; Xis, gene encoding excisionase.

**Table S3.** Primers used in this study

| Primer name | Primer sequence (5' to 3') |
| --- | --- |
| P-fw | GATTCCAGTAGCTAATTTCACACAGGAGACTGCCATGGATTTTCCCCAGCAGCTGGAAGCCTGCGTGAAACAGGCCAA |
| P-rv | GTGGTGATGATGCATGGCAGTCTCCTTGTGTGAAATTGTTATCCGCTCACGATTCCACACATTATACGAGCCGATGATTAATT |
| Lin-fw | AATCTGTACTTCCAGGGTTCCACCGCCGTGCCCTCTATGCCCA |
| Lin-rv | GTGGCGGCCGCTCTATTAGCTACTGGAATCATACAACATGGTTTT |
| Ls1 | ACGTTGTTGCCATTGCCCTGTTGACAATTAATCATCG |
| His-fw | ATGCATCATCACCACCATCAC |
| LIS-rv | TTAGCTACTGGAATCATACAACATGGT |
| Ldh-F | TTCTGTATTCAGACCGTCTACCTGATTCTATTAAATAAGGAAAAGATAAAGAAAGGTCATTTTTCCTGAATATGCTCACA |
| Ldh-R | ACAGCGCCACCTCGCCCGTTATCAGCCTGAAGAGAGTACAGCTCAAAAGGTCGTTTGTTGACAGCTGGTCCAATG |
| adhE-F | GGATTCAGGCTTGTTTACTAAAAAAAGTTTAACTTCCTCAGGAGAGCACAGAAAGGTCATTTTTCCTGAATATGCTCACA |
| adhE-R | ACGGGCCAGACAAGGGGTTTCGGCAGCCCGTTCATCGGGCGCGGAGCGGATCGTTTGTTGACAGCTGGTCCAATG |
| P1 | GAAGGAGATATAATGACGATGTGTTCAGCCCCCGGTAAGG |
| P2 | GCCAGTGAATTCTTATTGGATGAATATTCCCTCCGCCGTT |
| P3 | CATTATATCTCCTTCTTAAAGTTAAACAAA |
| P4 | TAAGAATTCACTGGCCGTCGTTTTACAACG |
| P5 | GGCCAGTGCCAAGCTTCTCGGTACCAGATCTCCCTGTTGA |
| P6 | CCTCTAGAGTCGACCTGCAGTGTAAAACGACGGCCAGTGAATTC |
| P11 | AATCTGTACTTCCAGGGT AGCACGGCGGTTCCCAGCATGCCCACC |
| P12 | GTGGCGGCCGCTCTATTATTTGTTACGCTGAATGATGTAGTCGGC |
| P13 | CAGCGTAACAAATAAACTCAACCAAGTCATTCTGAGAATAGTGTATGCGGCG |
| P14 | GTGGTGATGATGCATGGCAGTCTCCTTGTGTGAAATTGTTATCCGCTCACGATTCCACACATTATACGAGCCGATGATTAATT |
| P15 | TTATTTGTTACGCTGAATGATGTAGTCGGC |
| P16 | TGTGAAATTAGCTGCTGCTATCATACAGCA |
| P17 | GCAGCTAATTTCACACAGGAGACTGCCATGGACTTCCCCCAGCAG |
| P18 | ATGACTTGGTTGAGTTTATTTGTTACGCTGAATGATGT |
| P19 | GGAGATATACATATGTCCACCGCCGTGCCCTCTATGCCCA |
| pSOL-fw | CATATGTATATCTCCTTCTTATAGTTAAAC |
| pSOL-rv | TAATAGAGCGGCCGCCACCGCTGAGCAATA |
